# Neural Circuit and Clinical Insights from Intraoperative Recordings During Deep Brain Stimulation Surgery

**DOI:** 10.3390/brainsci9070173

**Published:** 2019-07-20

**Authors:** Anand Tekriwal, Neema Moin Afshar, Juan Santiago-Moreno, Fiene Marie Kuijper, Drew S. Kern, Casey H. Halpern, Gidon Felsen, John A. Thompson

**Affiliations:** 1 Department of Neurosurgery, University of Colorado School of Medicine, Aurora, CO 80203, USA; 2 Department of Physiology and Biophysics, University of Colorado School of Medicine, Aurora, CO 80203, USA; 3 Medical Scientist Training Program, University of Colorado School of Medicine, Aurora, CO 80203, USA; 4 Department of Neurosurgery, Stanford University School of Medicine, Stanford, CA 94305, USA; 5 Department of Neurology, University of Colorado School of Medicine, Aurora, CO 80203, USA

**Keywords:** deep brain stimulation, electrophysiology, intraoperative, human cognition, single-units, local field potentials, functional neurosurgery

## Abstract

Observations using invasive neural recordings from patient populations undergoing neurosurgical interventions have led to critical breakthroughs in our understanding of human neural circuit function and malfunction. The opportunity to interact with patients during neurophysiological mapping allowed for early insights in functional localization to improve surgical outcomes, but has since expanded into exploring fundamental aspects of human cognition including reward processing, language, the storage and retrieval of memory, decision-making, as well as sensory and motor processing. The increasing use of chronic neuromodulation, via deep brain stimulation, for a spectrum of neurological and psychiatric conditions has in tandem led to increased opportunity for linking theories of cognitive processing and neural circuit function. Our purpose here is to motivate the neuroscience and neurosurgical community to capitalize on the opportunities that this next decade will bring. To this end, we will highlight recent studies that have successfully leveraged invasive recordings during deep brain stimulation surgery to advance our understanding of human cognition with an emphasis on reward processing, improving clinical outcomes, and informing advances in neuromodulatory interventions.

## 1. Introduction

Currently, many tools are available to map the mind, and most studies of human cognition rely on non-invasive methods such as functional MRI (magnetic resonance imaging), MEG (magnetoencephalography), EEG (electroencephalography), and TMS (transcranial magnetic stimulation) to understand brain function. Beginning in the first quarter of the 20th century, with the use of EEG, scientists and clinicians sought to translate the language of the mind through electrical signals [1]. It was not until the early 1960s—with the introduction of microelectrode recordings—that single neuron recordings were possible [2]. The use of invasive, extracellular microelectrode recordings revolutionized the precision that could be obtained in stereotactic neurosurgical operations targeting deep brain nuclei [3,4]. Currently, invasive microelectrode recordings are used in the context of deep brain stimulation (DBS) implant surgeries and targeted ablative procedures for the treatment of movement disorders such as: Parkinson’s disease (PD), essential tremors, and dystonia [5,6,7]. DBS is Food and Drug Administration-approved for the treatment of PD, essential tremors, and epilepsy; it also has a humanitarian device exemption for the treatment of dystonia and obsessive-compulsive disorder (OCD), and is being investigated for no less than nine indications ranging from anorexia nervosa to tinnitus [8]. DBS involves the precise placement of a macroelectrode within the therapeutically specified region of a brain structure [9]. In the context of DBS surgeries, microelectrode recordings allow for the localization of a brain structure not easily visualized or one that juxtaposes brain structures that must be avoided to prevent unintended side effects [10,11]. The clinical application of single-unit microelectrode recordings allow brain mapping and target identification, and can account for both brain shift due to cerebrospinal fluid leak as well as magnetic imaging artifacts [12,13,14,15,16,17].

In addition to their clinical utility, recording from individual neurons of deep brain structures in an awake human provides the opportunity to explore experimental questions related to fundamental aspects of what it *means* to be human: perception, emotion, decision making, motor learning, and memory formation [18,19]. Although our understanding of human brain function and structure has increased with advances in functional imaging, neuronal dynamics underlying cognitive processes occur on a temporal scale that imaging technology cannot yet capture [20]. Single-unit electrophysiological parameters permit sub-millisecond sampling of neuronal processes; this is a temporal resolution that allows the correlation of spiking activity with behavioral responses [21]. Related signals called local field potentials (LFP) also provide high temporal precision and are more robust than single-unit recordings, but at the cost of lower spatial resolution [22]. For this reason, single-unit recordings have long been the gold standard in systems neuroscience for relating precise spike timing with aligned task events to understand brain function [20,23,24,25,26,27]. Nonetheless, LFP are an important complementary signal to single units, as LFP are easier to acquire, viable from chronically implanted probes, and shown to be particularly sensitive to picking up on synchronous activity such as oscillations [28,29,30]. Since both single-unit and LFP recording afford high temporal precision with other complementary advantages, each are leveraged during the course of clinical care, especially DBS surgeries.

DBS affords researchers the opportunity to study neural circuits that could not have been accessed two decades ago [7,8]. While exciting, this expansion is counterbalanced by the increased use of asleep intraoperative procedures, which are better tolerated by patients but limit the scope of intraoperative research. Similarly, the wireless stimulation of deep brain structures has already been proven in animal models, and if clinically implemented, it will remove the need for invasive probe placement [31]. These factors create an interesting situation for researchers, since as the complexity and novelty of intraoperative studies increases, the runway on such studies shortens. We have written this review in order to motivate the neuroscience and neurosurgical community to capitalize on the intraoperative opportunities this next decade will bring. To this end, we draw attention to recent intraoperative work that has advanced both clinical and basic science research, organized by recording signal (ECOG (electrocorticography), LFP, single unit; Table 1, Table 2 and Table 3).

The intraoperative studies we summarize span the range of DBS indications and stimulatory targets, excluding the ANT (anterior nucleus of the thalamus), which is a recently FDA-approved treatment of refractory epilepsy. As a newly approved target, studies relating features or domains of cognitive performance to this region of the thalamus have yet to be published. In addition to stimulatory targets themselves, all of the studies that we summarize report on the activity of regions that have an FDA–HDE approval for specific indications (i.e., NAc (nucleus accumbens)), and are regions that are passed through as part of the standard procedure that is used to gain access to clinically relevant targets, or define optimal therapeutic targets (i.e., SN). We draw attention to the function of these regions largely for their role in understanding human cognition as opposed to directly advancing patient care.

In the following sections, we have selected groups of studies that build off one another for further discussion. Broadly, the first half of this review relates specific deep brain structures’ function in human cognition, specifically reward-driven behavior. We have chosen to focus on reward-driven behavior, as dysregulation of satiation is a common refractory symptom in many neurological diseases. In addition, examining the biological basis of reward-driven behavior in humans benefits from the extensive work performed with the help of animal models. The latter half of our review discusses the clinical application of intraoperative electrophysiology in DBS procedures, focusing on how research conducted in the operating room can lead to near-term improvements in treatment efficacy. This organization is meant to impress upon readers the relevance of intraoperative research and provide readers with a map for what kinds of electrophysiology research will be possible in the future.

## 2. Reward Driven Behavior: NAc

The nucleus accumbens (NAc), as a key node of the mesocorticolimbic circuit, is a primary target for neuromodulation in the treatment of neuropsychiatric disorders such as addiction and OCD, which share a common feature: pathological impulsivity [68,69,70]. Situated at the interface between cortical and subcortical structures, the NAc integrates limbic, affective, and cognitive inputs in order to drive reward-guided behaviors. It is a crucial structure in reward valuation and action selection under conditions of uncertainty [38,71,72,73,74]. In this section, we provide an overview of the single-neuron and LFP studies investigating the neural processing of reward-guided behaviors in the human NAc, as well as a critical perspective on these electrophysiological contributions to our understanding of NAc reward-related signals.

To understand the basic mechanisms underlying the role of the NAc in decision making in the context of a potential reward, Patel et al. investigated single-neuron responses in the NAc of eight human subjects during a financial decision-making task [52]. For the experimental setup, a pair of playing cards was displayed to the subject on a computer screen, one face up to the subject and one face down intended for the computer opponent, with the highest card declared winner. Upon evaluating their card, the subject was asked to place a wager in order to maximize profit. The authors found that single neuron activity predicted future financial decisions on a trial-by-trial basis. This activity occurred approximately two seconds before subjects initiated their decision, which is a notably long latency between electrophysiologic signal and behavior. The authors reported that following feedback on whether their wager was won or lost, NAc activity was found to encode a prediction error signal, which was consistent with the previously described cell activity in related brain regions including the substantia nigra (SN) [19,75,76,77,78]. In comparing the timing of prediction error signals between the NAc and SN, Patel et al. found peak prediction error activity occurred at 250 ms and 450 ms, respectively, after receiving feedback, which is consistent with the time required for dopamine transmission from midbrain to striatal structures [52]. A notable characteristic of the NAc prediction error signal itself was that the increase in firing upon positive outcomes was proportionally greater than decreases for negative outcomes, indicating that perhaps the NAc is more responsive to rewards than losses [52]. Taken together, these findings highlight the importance of the NAc in the evaluation and execution of decisions in the context of reward.

Although the results from Patel et al. confirm the relevance of targeting the NAc with DBS in impulse control disorders, case studies suggest that therapeutic responsiveness to DBS treatments between patients is more variable than for movement disorders, which is often attributed to the insufficient spatial and temporal specificity of the current DBS procedure [71,72]. The NAc is a functionally heterogeneous structure that is divided into a presumed core and shell, which are differentially connected to the hubs of the reward circuitry [74]. In a study by Miller et al., intraoperative recordings were performed during DBS surgery for the treatment of OCD in a patient with cleanliness/contamination obsession. Specifically, the patient was given a toothbrush to hold to their face and asked to “imagine brushing your teeth with this dirty tooth brush” [43]. Interestingly, authors revealed a highly specific gamma-band oscillation in the NAc in response to an obsessive thought, as well as an increase in firing rate restricted to the dorsal part of the NAc. These findings constitute an initial step toward a better understanding of obsession processing in the human brain and shed light on the functional specificity of NAc subregions.

The NAc as a neuromodulatory target has notably benefited from translational research in recent years. In 2013, Halpern et al. found that delivering DBS to the NAc shell subregion during exposure to a food reward blocked binge eating in mice [73]. In a recent preclinical study building off this work, Wu et al. observed an anticipatory delta-band oscillation preceding a consummatory behavior in binge-like eating mice [71]. A responsive neurostimulation system allowed them to effectively detect and trigger the NAc after such an anticipatory signal, reducing consummatory behavior in mice sensitized to highly palatable food. To evaluate the translational potential of this finding, they recorded intraoperative NAc LFP in an OCD patient during a monetary-reward task, and observed a similar delta-band oscillation during anticipation of reward.

At a fundamental level, intraoperative electrophysiological recordings provide us with unique information on the role of the NAc in reward-guided behaviors, clarifying the neural basis of reward valuation and decision making under uncertainty, as well as the underlying temporal dynamics. By their contribution to the spatial and temporal optimization of NAc neurostimulation for impulse control disorders, LFP studies constitute a valuable tool for neurosurgical researchers.

## 3. Reward-Driven Behavior: GPi and STN

Reward-driven behavior depends upon an integrated network of distributed circuits including pathways originating from the dopaminergic (DAergic) neurons found in the NAc and ventral tegmental area, as well as a pathway from the internal segment of globus pallidus (GPi) to lateral habenula, as outlined by Hikosaka et al. [79,80,81,82]. Reward responsive neurons in this circuit include those that increase firing in response to loss or neutral outcomes, as well as neurons that increase firing in response to reward. In the last several years, researchers have begun to leverage access to the GPi in humans being treated with DBS, which has already been relatively well studied with respect to motor tasks to study non-motor, reward-related behavior.

In 2016, Howell et al. reported on findings from the GPi of eight patients during DBS for treatment of a range of indications, including PD, multiple system atrophy, cervical dystonia, and myoclonus dystonia [56]. Participants completed a reaction time task in which a button press was required after one of three visual stimuli were presented [83]. Stimuli corresponded to possible reward, no change, or a loss of digital currency that had no real-world value, with reward acquisition and loss avoidance possible if the button press was rapid enough. Of the 35 neurons recorded, two cells displayed a response to reward stimuli. The percent of neurons responsive to reward (5.7%) was much less than the percent of neurons responsive to movement (35%), but given the weak incentives motivating participants, a larger population may likely exist. Notably, these neurons did not increase firing for positive rewards, but rather for negative or null feedback, which is in line with findings from non-human primate GPi [80].

In a similar study, Rossi et al. investigated whether individual neurons in the GPi, as well as subthalamic nucleus (STN), responded to reward [57]. In this experiment, PD patients undergoing DBS surgery learned to associate one of four color patch visual stimuli with either reward for action, loss for action, reward for inaction, or loss for inaction. Actions were reported using a button press with rewards or losses equated with monetary gains or losses, respectively. Researchers recorded 100 STN cells from 20 patients and 100 GPi cells from 30 patients, finding that 70% of STN neurons and 46% of GPi neurons were responsive to the reward valence (i.e., for a given stimulus, whether association is a gain, loss, or null). STN neurons were more responsive to a potential reward than a potential loss, whereas GPi neurons were equally responsive. The authors posit that their findings can account for the greater incidence of impulse control disorders following STN compared to GPi DBS treatment, as the much smaller STN with its larger proportion of positive reward would presumably be more modulated than the GPi by equivalent intensity stimulation [84,85]. Drawing upon the animal literature, the authors observed that the distribution of reward-sensitive neurons in the GPi seemed uniform, in contrast to non-human primate findings that have implicated rostrally-located GPi neurons projecting to the lateral habenula in reward-related behavior [79,80,86,87]. This divergence may be due to differences in non-human primates compared to human physiology, or perhaps a result of PD pathophysiology in the human participants. Regardless, this finding also supports the lower incidence of impulse control disorders following GPi stimulation, as diffusely spread neurons would be less likely to be systemically modulated by locally applied stimulation [57].

## 4. Reward Driven Behavior: SN

The SN, a midbrain structure, is divided into at least two distinct regions based on anatomic and functional differences: the pars reticulata (SNr) and pars compacta (SNc) [88,89]. The SNr is one of two output nuclei of the basal ganglia, and similar to most basal ganglia nuclei, it is predominantly composed of fast spiking GABAergic neurons [90,91,92]. The SNr receives afferents from the STN as well as the striatum, and projects to the thalamic subnuclei, the superior colliculus, and motor output nuclei [90,93,94,95]. The SNc is the location of slow spiking, phasic firing, DAergic neurons, which are known to be affected in PD pathogenesis, and is strongly connected to the striatum, thalamus, and other basal ganglia subnuclei [88,96,97]. Functionally, the SN is thought to exert gain control over the internal drive for—and subsequent execution of—reward-seeking behavior [89,98,99]. More specifically, the SNr is associated with motor control, and the SNc is associated with reward expectation, with some overlap [76,89,100]. In order to understand the functional significance of these two populations, Ramayya et al. have investigated the composition and function of human SN using single-unit recordings and microstimulation to identify and selectively perturb GABAergic versus DAergic cells. Prior to their series of papers, the SN had been investigated as a DBS target for PD patients with resistant axial motor symptoms with muted beneficial effects, but few intraoperative studies had reported on SN function pertaining to human cognition [19,101,102].

In their first paper, Ramayya et al. microstimulated the SN of 11 patients undergoing DBS for the treatment of PD [55]. Although often used as part of the clinical procedure, the use of microstimulation in conjuncture with an intraoperative cognitive task was an inventive component of the authors’ experimental design. For their intraoperative task, patients were presented with a visual stimulus composed of two objects and asked to select one using a left or right button press. Following their choice, subjects received probabilistically determined positive or negative feedback. For a given pair of stimuli, one item would have a high probability of reward (>0.5), while the other would have a low probability (<0.5), with the sums equaling 1. Whether an item appeared on the left or right was randomly varied trial to trial, disentangling stimuli selection from directional button press. Microstimulation was applied during a subset of trials when feedback was being received with the hypothesis that stimulation would promote a repeating of the action, but not stimulus selection. The authors’ main finding was that microstimulation of the SN disrupted the learning of stimulus-reward associations when stimulation was paired with high but not low reward probability outcomes. Whether this was the result of impairment in learning or the development of an action-reward bias is unclear. Microstimulation was not cell type-specific, but recordings taken prior to microstimulation allowed post hoc categorization of SN neurons as GABAergic or DAergic based on the firing rate and waveform durations [103]. The authors found that generally, putative DAergic neurons demonstrated post-reward bursts that were consistent with a role in providing reward reinforcement. GABAergic neurons showed a wider range of firing patterns, with many demonstrating delayed and tonic increases in activity following positive feedback, and others engaging in long pauses.

Following up on their initial findings, Ramayya et al. published intraoperative findings using the same paradigm, but maintained stimulus-response mapping, i.e., a given visual stimuli would consistently appear in the same position, thereby causing stimulus–reward and action–reward associations to remain correlated. Leveraging the ability to identify putative GABAergic and DAergic cells based on single-unit characteristics, the authors expected microstimulation to improve learning when positioned near DAergic neurons, but induce impairments when near GABAergic neurons. Fitting with their hypotheses, the authors observed the greatest impairments in learning when stimulation was applied near putative GABAergic neurons. This finding, along with past work that could be interpreted to indicate microstimulation near DAergic neurons enhanced action–reward learning, present a convincing argument that these neuronal subpopulations have distinct and opposing functional roles [55,103].

The string of intraoperative papers by this group of researchers demonstrate that rigorous hypothesis-driven work can be carried out in the intraoperative setting. Furthermore, the authors’ experimental design and findings provide an invaluable bridge to work in rodent and non-human primate models.

## 5. Clinical Application: Improving DBS Outcomes with Electrophysiology

In recent years, it has been established that there are aberrant oscillatory signals within the GPi and STN that are unique to PD and dystonia [42,104]. The observation that the presence of these electrophysiological signals correlates with worsened symptoms has led to their investigation as potential biomarkers [105,106,107]. Current DBS systems are open-loop, meaning that the amount of stimulation delivered to targets is constant and not altered by any input signals. In a closed-loop DBS system, which is also referred to as adaptive DBS (aDBS), the timing of delivered stimulation is modulated based upon recordings of neuronal pathophysiological signals. The potential advantages of aDBS over conventional DBS systems are currently being investigated with the aim of providing greater clinical benefit with reduced adverse effects while utilizing less battery consumption [30,108,109,110]. Intraoperative studies are at the center of efforts to test such systems and elucidate the function of relevant circuitries.

In PD, it has been well described that increased beta activity (13–35 Hz) oscillations in the STN and GPi occur [42,111,112,113]. Beta activity is thought to normally be indicative of a resting state; however, in PD, an increase in beta activity results in worsened symptoms, particularly bradykinesia and freezing of gait [42,107,111,112]. With the administration of DAergic medications, the power spectrum changes from heightened beta-band power to decreased beta-band power along with an increase of gamma-band (>35 Hz) power [107]. Although not all beta activity is representative of pathophysiology, in PD, it appears that longer duration beta signals (e.g., >0.6 s) are correlated with worsened motor function compared with short bursts of activity [111]. Interestingly, and intuitively, a greater duration of beta bursts has been correlated with greater amplitude [111]. In a recent study comparing intraoperatively recorded LFP from the STN and GPi, Pine-Fuentes et al. reported no significant difference in parkinsonian beta signal oscillations, including the burst characteristics and variation of beta oscillations, between the two nuclei [42]. These findings highlight the robustness of beta activity as a real-time biomarker for the PD symptomatic state. Adaptive DBS that is responsive to changes in beta amplitude has been applied with promising results in PD, resulting in the reduction of longer duration beta bursts and of higher beta amplitudes compared with conventional DBS [111]. Studies have also demonstrated clinical benefits of aDBS with mitigated stimulation-induced adverse effects including dysarthria and levodopa-induced dyskinesias [107].

For the management of specific features of PD, other oscillatory bands, including gamma, have shown value [114,115]. Interestingly, pilot data has indicated that *greater* gamma oscillations may be correlated with dyskinesias within the STN and motor cortex [116]. In order to investigate whether the hyper-direct pathway may be the cause for coherence between these two areas, Miocinovic et al. stimulated DBS electrodes placed within the STN or GPi and recorded evoked potentials in the sensorimotor cortex via a subdural strip of electrodes [32]. The authors reported observing evoked potentials when stimulating through certain contacts of DBS electrodes within the STN, but not GPi. Notably, contacts that were found to evoke cortical potentials were predictive of clinical benefit, potentially reflecting the antidromic stimulation of the corticosubthalamic hyperdirect pathway [32].

In dystonia, the identification and use of oscillatory markers has benefited from lessons learned in the study of PD. Analogous to beta oscillatory activity in PD, a greater amplitude of low-frequency bands (4–12 Hz) have been recorded in the GPi of dystonic patients and have been shown to have coherence with the EMG (electromyography) of dystonic limbs [117,118]. In their 2019 study, Pina-Fuentes et al. also recorded from and applied aDBS to the GPi of dystonia patients in addition to the PD patients as described above. The authors reported that theta oscillatory activity of 4–7 Hz within the GPi positively correlated with worsened dystonia symptoms, as expected [42]. Furthermore, with aDBS, there was a reduction of theta oscillatory activity and coherence to muscle activity recorded by EMG. Interestingly, although no clinical evaluation was performed while aDBS was applied for the treatment of dystonia, the patient reported a subjective relief of symptoms [42].

With the advancement of technology, novel DBS systems with sensing capabilities are currently being developed. Overall, adaptive stimulation appears safe with minimal adverse effects, although inadvertent muscle contractions have been reported, which are likely due to the transient spread of stimulation to the internal capsule. Future directions will require the determination of the exact phase of signal to deliver stimulation, as well as the form of stimulation that simultaneously reduces the pathophysiological signal while allowing for non-pathological signals within the neuronal network [119]. In the near future, aDBS systems will become commercially available, and clinicians will need to gain expertise in this form of stimulation to maximize clinical benefits.

## 6. Clinical Application: Intraoperative Targeting with LFP

The standard approach for DBS implantation, in many centers around the world, relies on the interpretation of single-unit microelectrode recordings for targeting the brain region of interest [17]. This method, while being the standard of care, is limited both by the experience and ability to recognize patterns that allow for the accurate anatomical mapping of these regions. The use of LFP offers a complementary measure of neuronal activity that may help localize probe placement, and in contrast to single units, can be recorded from the implanted stimulatory macroelectrode and require less technical expertise to interpret [28,120].

Recent efforts have sought to use LFP features to automate the localization of STN and identify the optimal stimulation target [9,121,122,123]. In one study designed to assess whether LFP could add information to improve treatment outcomes, Telkes et al. used STN LFP recorded from 22 PD patients to predict the optimal track for stimulation [39]. Using LFP recorded during three mapping trajectories, researchers attempted to predict the track ultimately used by the neurosurgeon. Using only beta-band or high-frequency oscillations provided prediction accuracies of 72% and 68% respectively; however, by analyzing the combined data from both high and low-frequency bands, researchers achieved an 80% prediction rate. This study provided some of the first evidence that LFP could be leveraged in the operating room. Recently, the clinical utility of LFP was expanded upon with another paper by Telkes et al. in which subtypes of PD, such as tremor dominant and postural instability with gait difficulty, were distinguished based on an analysis of LFP recorded from multiple microelectrodes placed in the STN [124]. Authors reported that coupling between recorded beta band and either slow or fast high-frequency oscillations (200–260 Hz, 260–450 Hz respectively) can be used to differentiate PD subtypes, which is an interesting finding that will likely contribute to the personalization of DBS treatments.

## 7. Clinical Application: Use of Electrophysiology During Asleep DBS

Advances in neuroimaging technology have made possible the use of “asleep” DBS where the targeting and implantation of DBS electrodes is performed while patients are under general anesthesia [125,126]. This approach relies on the direct visualization and targeting of nuclei in the basal ganglia [127]. Intraoperatively, lead placement is confirmed using either intraoperative CT or intraoperative MRI (iMRI). Multiple reviews have confirmed its comparable efficacy in terms of symptom improvement and long-term reductions in levodopa dosage between awake and asleep DBS in the STN and GPi [125,126,128]. However, studies examining iMRI data have noted the potential for substantial and unpredictable brain shift following the dural opening [12,13,14,15,16]. Inaccurate targeting as a result of brain shift may lead to inaccurate electrode placement and a higher risk of stimulating the wrong region, thus eliciting undesirable off-target effects. For these reasons, a hybrid approach is being explored where electrophysiological targeting is added to the asleep DBS approach. However, this is not without challenges. General anesthesia is widely accepted to be associated with a decreased neuronal spiking rate in the STN of DBS patients, although this has been debated [129,130]. LFP appear to be more susceptible to anesthesia, as a recent study by Malekmouhammadi et al. has demonstrated diminished relative power differences in beta oscillation and high-frequency oscillation in LFP recorded from the GPi of asleep patients compared to awake patients [131]. Authors reported that the differences in LFP between the GPe and GPi were all but obliterated under general anesthesia, severely limiting the potential of LFP as an aid in intraoperative targeting [131].

## 8. Conclusions

Single-neuron and LFP recordings in awake human subjects have afforded unique opportunities to improve the application of neuromodulatory therapy and surgical targeting while also enhancing our understanding of deep brain nuclei’s cognitive functional significance. Hardware and programming software for DBS therapy have recently experienced a significant increase in the rate of technological advance, from new electrode designs to semi-automated post-operative programming platforms, and the potential to improve upon the efficacy of conventional DBS is real, with implications for research just starting to be realized. Specifically, intraoperative and perioperative experiments have directly contributed to the near future implementation of aDBS. In contrast, if asleep DBS procedures become the standard, then it is likely that the neuroscience-oriented studies that we have highlighted in this review will become increasingly rare. Quantifying the benefits of clinical and basic science advances due to such studies is exceedingly difficult, but should be considered as the practice of stereotactic and functional neurosurgery evolves.

## Figures and Tables

**Table 1:** ECOG: electrocorticography. DBS: deep brain stimulation, GPi: globus pallidus, PD: Parkinson’s disease, STN: subthalamic nucleus.

Year	Author(s)	Journal	DBS Indication; Location	Summary
**2018**	Miocinovic et al. [32]	The Journal of Neuroscience	PD; STN (9)	Intraoperative ECoG recordings from subdural cortical areas demonstrate short-latency evoked potentials arising from STN stimulation that were distinct from very short-latency evoked potentials arising from corticospinal and corticobulbar tracts. No short-latency evoked potentials were seen from GPi stimulation. These potentials are indicative of a hyperdirect pathway between the STN and cortex.

**Table 2:** Local field potentials (LFP). EEG: electroencephalography.

Year	Author(s)	Journal	DBS Indication; Location	Summary
**2002**	Williams et al. [33]	Brain	PD; STN (13), GPi (2)	Simultaneous LFP and EEG recordings illustrate a complex frequency-dependent topography in basal–cortical connections with the basal ganglia receiving multiple inputs at frequencies <30 Hz and driving connection to the cortex at higher frequencies (~70 Hz).
**2005**	Wingeier et al. [34]	Experimental Neurology	PD; STN (6)	The intraoperative recordings of LFP within the STN demonstrated a reduction in the power of the beta band, which also persisted briefly after DBS was discontinued.
**2005**	Kühn et al. [35]	Experimental Neurology	PD; STN (8)	Simultaneous intraoperative LFP and microelectrode recordings found that the beta-band LFP activity that is generated from within the STN and LFP oscillations seemed to be generated through the synchronization of local neuronal activity.
**2006**	Weinberger et al. [36]	Journal of Neurophysiology	PD; STN (28)	Simultaneous LFP and single-unit recordings illustrate that the degree of neuronal beta oscillatory activity is correlated to the responsiveness to dopamine medication, as well as reinforcing that beta oscillatory neurons are found in the dorsal STN.
**2010**	Hirschmann et al. [37]	Neuroimage	PD; STN (8)	Simultaneous LFP and MEG recordings revealed frequency-related interactions among the STN and cortex in the beta and alpha frequencies.
**2012**	Abosch et al. [29]	Neurosurgery	PD; STN (9)	LFP recordings conducted intraoperatively, post-operatively (3 weeks), and 2 to 7 years post-surgery demonstrate similar recordings with slight decreases in beta activity 2 to 7 years after surgery.
**2016**	Stenner et al. [38]	Journal of Neurophysiology	Epilepsy; NAc (8)	LFP recordings of the nucleus accumbens (NAc) of patients during a decision-making task resulted in a decrease of beta power prior and post-execution of action. These results help imply that the NAc is involved in action preparation.
**2016**	Telkes et al. [39]	Frontiers in Neuroscience	PD; STN (22)	Comparison of intraoperative recordings of LFP and microelectrode recordings resulted in evidence that LFP can be a viable marker in guiding the macroelectrode during surgery.
**2017**	Kolb et al. [40]	Physiological Reports	PD; STN (21)	Intraoperative LFP recordings illustrate that deep brain structures can be differentiated via the comparison of high beta band activity. Other frequencies can aid in the differentiation of the striatum from the thalamus and STN.
**2018**	Wang et al. [41]	The Journal of Neuroscience	PD (20), Dystonia (14), GPi	Intraoperative recordings of PD patients were compared with dystonic patients. Results demonstrate elevated synchronization between the beta band and motor cortex within PD, while the elevated theta band was seen in dystonic patients. These results indicate distinct frequency synchronization between various movement disorders.
**2019**	Pīna-Fuentes et al. [42]	Neurobiology of Disease	PD and Dystonia; STN(6), GPi (12)	Intraoperative LFP recordings in the GPi and STN for PD and dystonia were measured to uncover differences and similarities in the beta band frequency. Results demonstrate that frequency markers differ between diseases; however, spectral frequency is similar between nuclei in the same disease, which may aid in adaptive DBS.
**2019**	Miller et al. [43]	Journal of Neurophysiology	OCD; Nucleus Accumbens (1)	Intraoperative LFP were recorded from an awake OCD patient. Researchers were able to measure oscillatory activity upon the onset of obsession; the recordings demonstrate a modulation of firing and amplitude within the gamma band.

**Table 3:** Single Unit. OCD: obsessive-compulsive disorder, MRI: magnetic resonance imaging.

Year	Author(s)	Journal	DBS Indication; Location	Summary
**1964**	Gaze et al. [2]	Brain	PD; Thalamus	Intraoperative single-unit recordings of the thalamus were conducted in PD patients to illustrate its ability to identify various structures and accurately determine the location of the electrode in DBS.
**2000**	Magnin et al. [44]	Neuroscience	PD; GPi; and Thalamus (29)	Single-unit recordings of the GPi and thalamus of 29 PD patients were recorded. The results indicate that the GPi has a faster firing rate than the GPe (globus pallidus externus), as well as demonstrating that the thalamus has a high proportion of units with low-threshold bursting activities. These results also support the view that PD symptoms arise from the low-threshold bursting and oscillatory activity in the thalamus.
**2000**	Levy et al. [45]	The Journal of Neuroscience	PD; STN (9)	Single-unit recordings from the STN of PD patients exhibiting tremor or non-tremor symptoms resulted in high-frequency oscillatory activity being prominent in patients with tremors, allowing for the association of such synchronous activity in tremor cells and limb tremors.
**2001**	Rodriguez-Oroz et al. [46]	Brain	PD; STN (14)	Intraoperative single-unit recordings of 350 neurons were conducted to create a somatotopic organization in the STN. The results concluded that the sensorimotor region was located dorsolaterally within the STN.
**2002**	Benazzouz et al. [47]	Movement Disorders	PD; STN (153)	Intraoperative single-unit recordings conducted from five microelectrodes resulted in the identification of STN from surrounding structures, i.e., SNr (substantia nigra pars reticulata), on the basis of firing pattern. Results also suggest different modes of firing and its association with parkinsonian symptoms.
**2002**	Abosch et al. [48]	Journal of Neurosurgery	PD; STN (70)	Retrospective analysis of intraoperative single-unit recordings demonstrated a clustering of movement-related receptive fields in the rostrodorsal region of the STN.
**2005**	Hamani et al. [49]	Surgical Neurology	PD; STN (18)	Accuracy of positioning in DBS was compared using the microelectrode recordings and MRI imagining. The results indicate that a good correlation exists; however, this correlation is not so strong in the anterior–posterior axis, as microelectrode recordings show the STN being more anterior than defined by the MRI.
**2009**	Shrock et al. [50]	Journal of Neurophysiology	Dystonia; STN (9)	Intraoperative single-unit recordings in patients with dystonia were compared to identical recordings in patients with PD. The results illustrate similar bursting and oscillatory activity, whereas oscillatory activity in dystonia is seen at lower frequencies than PD.
**2012**	Sarma et al. [51]	Frontiers in Integrative Neuroscience	PD; STN (7)	Intraoperative single-unit recordings of PD patients illustrate that beta oscillations are suppressed in the presence of a predicted *go* cue or internally generated *go* cue.
**2012**	Patel et al. [52]	The Journal of Neuroscience	OCD (3), MDD (major depressive disorder) (5); NAc (8)	Intraoperative single-unit recordings of the NAc during a financial decision-making task resulted in three key findings: 1. The NAc predicts the future financial choices, 2. The signal manifested 2 s prior to when the decision was made, and 3. NAc codes for a prediction error.
**2012**	Sheth et al. [53]	Nature	OCD; dACC (dorsal anterior cingulate cortex) (6)	Intraoperative single-unit recordings during a cingulotomy of the dACC demonstrates that the region contains neuronal activity for behavioral adaptation. The dACC will produce signals for efficiency in situations with stable difficulty or produce signals of latency in situations with varying difficulties.
**2013**	Guo et al. [54]	Parkinsonism and related Disorders	PD; STN (23)	Intraoperative recordings identified two oscillatory neurons (ßFB and TFB) localized in the dorsal STN.
**2014**	Ramayya et al. [55]	The Journal of Neuroscience	PD; Substantia Nigra (11)	Intraoperative microstimulation of the substantia nigra (SN) during a two-alternative probability learning task demonstrates decreased learning on reward trials with SN stimulation when compared to controls.
**2016**	Howell et al. [56]	Neuroscience	Multiple; GPi (8)	Intraoperative single-unit recordings of the GPi in patients with different indications resulted in findings that support the idea that non-motor information, such as reward information and visual stimuli, are carried in the GPi.
**2017**	Rossi et al. [57]	Human Brain Mapping	PD; STN; and GPi (50)	Single-unit recordings conducted during a reward/loss paradigm resulted in evidence that the STN and GPi both encode valence-related information with a higher proportion of these neurons found in the STN.
**2017**	Ramayya et al. [58]	Frontiers in Human Neuroscience	PD; SN (11)	Microstimulation of GABAergic neurons in the SN during a two-alternative reinforcement learning task led to an impairment in learning.
**2018**	Swan et al. [59]	Brain Stimulation	Essential tremor; ventral intermediate thalamus (VIM) (11)	Microstimulation of neurons within the VIM was conducted during DBS surgery. Stimulation was able to create sensory precepts on separate digits with variable intensity through adjustments of DBS resistance.
**2018**	Whatley et al. [60]	Journal of Neurophysiology	Essential tremor; nucleus of the ventral intermediate thalamus (VIM) (1)	Researchers were able to represent functional plasticity within the VIM of a patient who underwent bilateral amputation from the elbow down earlier in life. The patient, years after amputation, received DBS surgery during which microelectrode recordings demonstrated increased firing by VIM neurons in response to shoulder protraction. The VIM typically encodes hand movements; however, due to the amputation and the use of the shoulders to operate the prostheses, remapping was seen in the nucleus.
**2018**	Tankus et al. [61]	Journal of Neurosurgery	PD; STN (10)	Single-unit recordings were obtained from the STN of PD patients undergoing DBS surgery. Patients were asked to perform repetitive hand and foot movements at varying paces. The recordings demonstrate individual schemes for recruiting STN neurons controlling the upper versus lower extremities, with firing rates varying as the pace of movement was altered.
**2018**	Perez et al. [62]	Journal of Neurosurgery	Tinnitus; Caudate nucleus (6)	Direct electrical stimulation of the caudate nucleus was performed during DBS surgery for tinnitus loudness modulation. Results from the trials demonstrate a greater effect on reduction when stimulation occurred in the caudate body compared to the head. fMRI illustrated greater connectivity between the caudate body and auditory cortex when compared to the caudate head and auditory cortex.
**2018**	Myrov et al. [63]	Neuroscience Research	PD; STN (8)	Intraoperative recordings were conducted on awake or on patients under propofol-induced general anesthesia to discern various neuronal characteristics between these two states. The data demonstrates that STN neurons under general anesthesia have greater bursting, while witnessing a significant decrease in firing rate when compared to the awake counterparts.
**2018**	Luo et al. [64]	The Journal of Neuroscience	PD (8), Dystonia (4); GPi	High-frequency stimulation of GPi neurons during DBS surgery was conducted while neuronal firing was recorded simultaneously to identify the mechanism of after-facilitation seen in therapeutic DBS. The results of human intraoperative recordings and slice recordings from rodents demonstrate a multisynaptic mechanism consisting of glutamatergic, GABAergic, and cholinergic synapses modulating short and long-term effects of DBS in the GPi.
**2018**	Lipski et al. [65]	The Journal of Neuroscience	PD; STN (12)	Intraoperative single units were recorded in the STN of PD patients undergoing DBS surgery. Patients were asked to perform a speech task in which single syllables were presented and were asked to repeat the syllables. The results were able to demonstrate a functional connection between speech production and neuronal firing rates within the STN.
**2019**	Wenzel et al. [66]	Cell Systems	Epilepsy; anterior middle temporal gyrus (2)	Researchers conducted single-unit recordings during various anesthetic levels induced through propofol to discern cortical circuit level changes that lead to a loss of consciousness (LOC). The results illustrate a loss of discriminable microstates as well as a loss of neuronal ensembles, which contribute to LOC.
**2019**	Lee et al. [67]	Journal of Neurosurgery	PD; GPi; and nucleus basalis of Meynert (nBM) (5)	Intraoperative single-unit recordings were obtained during a resting state compared to an auditory attention task. Results demonstrate altered firing patterns in the nBM neurons, while GPi neurons remained consistent between trials. Such findings may aid in establishing the nBM as a therapeutic model for cognitive impairment in PD.
